# In Vivo Corneal Confocal Microscopy Detects Improvement of Corneal Nerve Parameters following Glycemic Control in Patients with Type 2 Diabetes

**DOI:** 10.1155/2018/8516276

**Published:** 2018-06-24

**Authors:** Xiaofan Jia, Xiaogang Wang, Xiaoxia Wang, Qi Pan, Tongzhang Xian, Xiaobin Yu, Lixin Guo

**Affiliations:** ^1^Department of Endocrinology, Beijing Hospital, National Center of Gerontology, Beijing, China; ^2^Department of Chinese Traditional Medicine, Beijing Hospital, National Center of Gerontology, Beijing, China; ^3^Department of Ophthalmology, Beijing Hospital, National Center of Gerontology, Beijing, China

## Abstract

**Aim:**

This study aimed to investigate whether in vivo corneal confocal microscopy (CCM) can detect the improvement of corneal nerve parameters following glycemic control in patients with type 2 diabetes in natural history.

**Methods:**

Thirty-two patients with diabetes complicated by DPN and 12 age-matched control subjects underwent detailed clinical examination and were assessed per the Toronto Clinical Scoring Scale for DPN, nerve conduction studies, and IVCCM at baseline and after approximately one year from the first visit.

**Results:**

At follow-up, 16 diabetic patients had improved glycemic control (group A, HbA1c < 7.0%, 7.78 ± 1.62% versus 6.52 ± 0.59%, *P* = 0.005), while the remainder continued to have elevated HbA1c levels (group B, HbA1c ≥ 7.0%, 8.55 ± 1.57% versus 8.79 ± 1.05%, *P* = 0.527). For patients in group A, corneal nerve fiber density (CNFD) (18.55 ± 5.25 n/mm^2^ versus 21.78 ± 6.13 n/mm^2^, *P* = 0.005) and corneal nerve fiber length (CNFL) (11.62 ± 2.89 mm/mm^2^ versus 13.04 ± 2.44 mm/mm^2^, *P* = 0.029) increased significantly compared to baseline. For patients in group B, sural sensory nerve conduction velocity (47.93 ± 7.20 m/s versus 44.67 ± 6.43 m/s, *P* = 0.024), CNFD (17.19 ± 5.31 n/mm^2^ versus 15.67 ± 4.16 n/mm^2^, *P* = 0.001), corneal nerve branch density (19.33 ± 12.82 n/mm^2^ versus 14.23 ± 6.56 n/mm^2^, *P* = 0.033), and CNFL (11.16 ± 2.57 mm/mm^2^ versus 9.90 ± 1.75 mm/mm^2^, *P* = 0.011) decreased significantly.

**Conclusions:**

The results of this study suggest that morphological repair of corneal nerve fibers can be detected when glycemic control improves. In vivo CCM could be a sensitive method that can be applied in future longitudinal or interventional studies on DPN.

## 1. Introduction

Diabetic polyneuropathy (DPN) is one of the most common chronic complications of diabetes. Over 50% of diabetic patients develop DPN as their disease course progresses [1]. Diabetic neuropathy leads to morbidity in diabetic patients in the form of painful neuropathy and foot ulceration with consequent lower limb amputation [2]. It accounts for reduced quality of life and imposes a significant economic burden on both individuals and society [3]. Prospective studies on diabetic neuropathy have revealed that neuropathy progresses gradually with time. A long-term follow-up study on type 2 diabetes reported that the rate of abnormal nerve conduction velocity (NCV) results was 8% at baseline, and it increased to 16% and 42% after 5 years and 10 years, respectively [4].

Metabolic factors including blood glucose, blood lipids, blood pressure, and body mass index (BMI) have been identified as risk factors for DPN [5]. However, clinical intervention studies correlating stricter control of such metabolic factors have rarely shown a corresponding improvement in DPN. The Epidemiology of Diabetes Interventions and Complications (EDIC) study showed that improved glycemic control in patients with type 1 diabetes yielded immediate and long-term benefits for DPN [6]. However, the Veterans Affairs Diabetes Trial (VADT) showed no obvious improvement in diabetic neuropathy resulting from glycemic control in patients with type 2 diabetes; further, it revealed a trend towards deterioration of autonomic neuropathies in the study cohort [7]. In the Steno-2 study, although targeted interventions aimed at multiple risk factors in patients with type 2 diabetes improved diabetic retinopathy, diabetic nephropathy, and cardiovascular autonomic neuropathy, similar improvements were not observed for somatic neuropathy [8].

The prevailing “mainstream opinions” in the medical community, therefore, reflect the belief that the neurologic impairments caused by diabetes are difficult to reverse. However, inconclusive results regarding neurological improvement of DPN may not reflect irreversibility but rather may stem from the lack of appropriately sensitive and effective evaluation methods that can be utilized to reveal the effect of glycemic control on DPN. Growing evidence supports a prominent association between corneal nerve morphology measured using corneal confocal microscopy (CCM) and DPN [9, 10]. As a quick, noninvasive, and reiterative technique, CCM has demonstrated the capacity to detect early small nerve fiber damage in diabetic patients [10, 11] and to diagnose [11] and classify the severity of DPN [12, 13].

Our study sought to determine whether improved glycemic control at one-year follow-up improved corneal nerve morphology and DPN in patients with type 2 diabetes.

## 2. Materials and Methods

### 2.1. Subjects

From September 2015 to March 2016, 60 patients (age range 30–80 years) with type 2 diabetes and HbA1c ≥ 7.0% were recruited. Type 2 diabetes was diagnosed according to World Health Organization criteria [14]. All patients received NCV testing; 50 were diagnosed with DPN owing to abnormal NCV results and symptoms or signs of DPN [15]. Thirty-two patients with DPN completed one-year follow-up, and 18 patients were lost to follow-up.

All patients were provided medical recommendations for antidiabetic, antihypertensive, and lipid-lowering therapy at the beginning of the study. During the next year, there were no limitations imposed on the patients regarding diabetic care. After one year had elapsed, the 32 diabetic patients who were followed up were divided into two groups (groups A and B) according to their HbA1c status relative to the control goal suggested by the Chinese Diabetes Society (CDS), which recommends an HbA1c < 7.0%. Sixteen individuals achieved an HbA1c < 7.0% and were assigned to group A, whereas group B was comprised of 16 patients who had not reached the HbA1c goal (i.e., HbA1c ≥ 7.0%) at one-year follow-up. Twelve age-matched, healthy volunteers without diabetes mellitus, prediabetes, and/or clinical or paraclinical signs or symptoms of polyneuropathy were recruited to form a control group.

Exclusion criteria were diseases affecting the central or peripheral nervous systems, malignant tumors, connective tissue diseases, acute and chronic hepatic or renal diseases, thyroid diseases, endocrinopathies, metabolic derangements, psychological conditions, diabetic foot ulcers, active oculopathy, history of ocular operation, glaucoma, acute and chronic corneal diseases, and an extended history of corneal contact lens use. Both diabetic and control participants were comprehensively examined before recruitment into the study to ascertain health status and ensure that no exclusion criteria were met.

The study was approved by the ethics committee of Beijing Hospital, and written informed consent was obtained according to the Declaration of Helsinki.

### 2.2. Clinical Assessment

All study participants underwent medical and neurologic assessments at baseline and at one-year follow-up (15.2 ± 1.6 months). Medical assessments included the measurement of systolic (SBP) and diastolic blood pressure (DBP), HbA1c (%), and lipid fractions (concentrations of total cholesterol (TC) (mmol/L), high- (HDLC) and low-density lipoprotein cholesterol (LDLC) (mmol/L), and triglycerides (TG) (mmol/L)).

### 2.3. Peripheral Neuropathy Assessment

The Toronto Clinical Scoring System (TCSS) for DPN was used [16]. Electrodiagnostic studies were performed using a Medoc “Key point” system (Medoc Dynamics Ltd., Bristol, UK). Tibial (TMNCV) and peroneal motor nerve conduction velocity (PMNCV), as well as sural (SSNCV) and superficial peroneal sensory nerve conduction velocity (SPSNCV), was measured in the left lower limb (calf-to-ankle) by a consultant neurophysiologist.

### 2.4. In Vivo Corneal Confocal Microscopy

All study subjects underwent examination with the Heidelberg retina tomograph-II in vivo corneal confocal microscope. The subjects' eyes were anesthetized using one drop of 0.4% benoxinate hydrochloride, and Viscotears were applied to the front of the eye for lubrication. One drop of viscoelastic gel was placed on the tip of the objective lens, and a sterile disposable Perspex cap was placed over the lens allowing optical coupling of the objective lens to the cornea. The patient was instructed to fixate the eye not being examined on a target. Several scans of the entire depth of the cornea were recorded by turning the fine focus of the objective lens backwards and forwards for ~2 min using the section mode, which enables manual acquisition and storage of single images of all corneal layers. This provided en face two-dimensional images with a lateral resolution of ~2 mm/pixel and final image size of 400 × 400 pixels of the subbasal nerve plexus of the cornea from each patient and control subject.

One examiner masked from patients' HbA1c results selected and analyzed 3 to 6 high-clarity images from the central subbasal nerve plexus. Criteria for image selection were depth, focus, position, and contrast. The examiner quantified the images with semiautomated, purpose-written, proprietary software (ACCMetrics, M. A. Dabbah, Imaging Science Biomedical Engineering, University of Manchester, Manchester, UK). Three corneal nerve parameters were quantified: (1) corneal nerve fiber density (CNFD), calculated as the total number of major nerves per square millimeter of corneal tissue (n/mm^2^); (2) corneal nerve branch density (CNBD), calculated as the number of branches emanating from all major nerve trunks per square millimeter of corneal tissue (n/mm^2^); and (3) corneal nerve fiber length (CNFL), calculated as the total length of all nerve fibers and branches within the area of the corneal tissue (mm/mm^2^).

### 2.5. Statistical Methods

SPSS 23.0 for Windows was used to compute the results. Analysis included descriptive and frequency statistics. All data are expressed as means ± standard deviation (SD). Independent-sample *t-*tests were used to test whether a sample mean differed between control subjects and diabetic patients. Paired-sample *t*-tests were used for the comparison between baseline and follow-up data. Nonparametric data were analyzed using *χ*^2^ tests. Pearson correlation analysis and linear regression were adapted for the correlation between corneal nerve parameters and other indexes. Statistical significance was set at *P* < 0.05.

## 3. Results

### 3.1. Comparison of Control and Diabetes Groups

Table 1 shows the clinical characteristics of control subjects and patients with diabetes at baseline and one-year follow-up. Notable differences in baseline characteristics between the groups included a higher HbA1c, SBP, and DBP in diabetic patients. Conversely, controls had significantly higher HDLC. When surveying TCSS, nerve conduction, and corneal nerve parameters, there were also significant differences between the two groups. TCSS was significantly lower in diabetic patients. All motor and sensory nerve conduction velocities were significantly slower in diabetic patients than in controls; similarly, diabetics' corneal parameters were significantly lower.

The control group showed no significant changes between examinations at baseline and follow-up. In the diabetic cohort, a significant improvement in glycemic control was demonstrated from baseline to follow-up HbA1c. Conversely, there were no significant changes in SBP and DBP, as well as levels of TC, TG, LDLC, and HDLC (Table 1). TCSS did not show a significant change at one-year follow-up. There was a significant decrease in conduction velocity seen in the PMNCV, TMNCV, and SPSNCV examinations; SSNCV examination did not yield a significant change at one-year follow-up.

### 3.2. Comparison of Diabetes Groups A and B

Table 2 shows the clinical characteristics of group A and group B at baseline and one-year follow-up. At baseline, there were no significant differences among the groups across all parameters.

**Figure 1:**
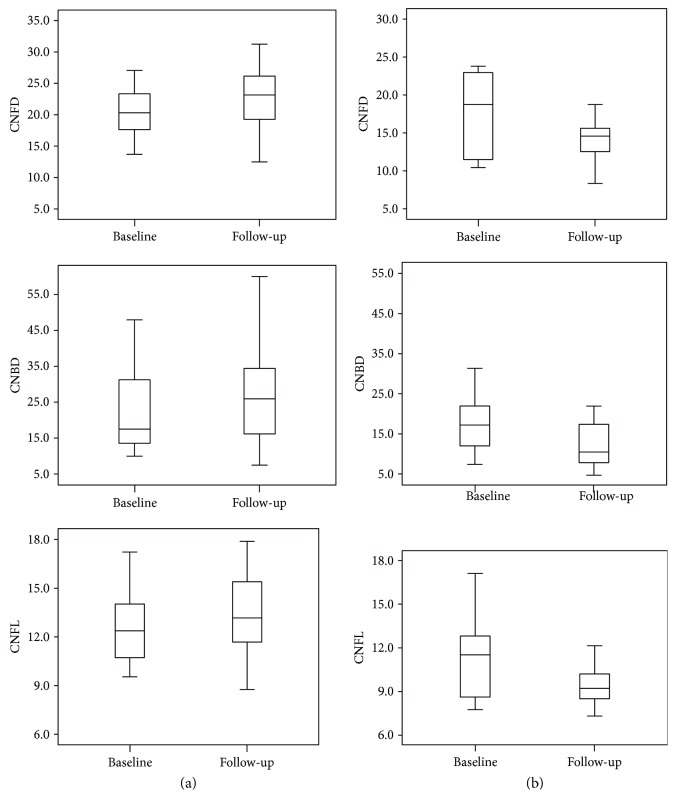
Changes of CCM (from top to bottom are CNFD, CNBD, and CNFL) from baseline to follow-up in group A (a) and group B (b).

**Figure 2:**
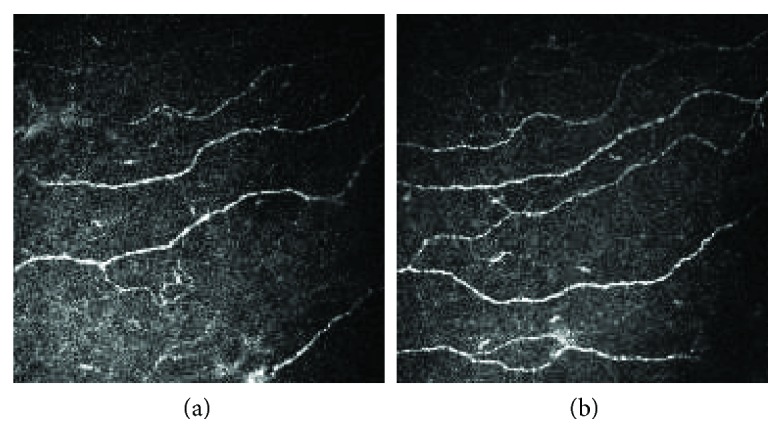
CCM images from a diabetic patient of group A at baseline (a) and follow-up (b).

At one-year follow-up, group A showed a significant decrease in HbA1c compared to baseline (from 7.78 ± 1.62% to 6.52 ± 0.59%, *P* = 0.005). No significant changes were seen for metabolic indexes (weight, SBP, DBP, TC, TG, LDLC, and HDLC) at the follow-up. For DPN assessment, no significant changes were observed for TCSS (4.4 ± 4.4 to 4.5 ± 4.8, *P* = 0.544) and NCV (TMNCV (44.15 ± 4.86 m/s to 43.56 ± 4.86 m/s, *P* = 0.269), PMNCV (44.47 ± 4.10 m/s to 44.46 ± 4.33 m/s, *P* = 0.984), SSNCV (48.87 ± 7.89 m/s to 47.28 ± 6.05 m/s, *P* = 0.293), and SPSNCV (50.42 ± 6.81 m/s to 50.14 ± 7.19 m/s, *P* = 0.287)), otherwise corneal nerve showed a significant improvement (CNFD (18.55 ± 5.25 n/mm^2^ to 21.78 ± 6.13 n/mm^2^, *P* = 0.005, change of 2.35 ± 3.53), CNBD (21.76 ± 16.10 n/mm^2^ to 26.19 ± 13.87 n/mm^2^, *P* = 0.122, change of 3.06 ± 12.31), and CNFL (11.62 ± 2.89 mm/mm^2^ to 13.04 ± 2.44 mm/mm^2^, *P* = 0.029, change of 0.90 ± 2.42)) (Figures 1 and 2).

**Figure 3:**
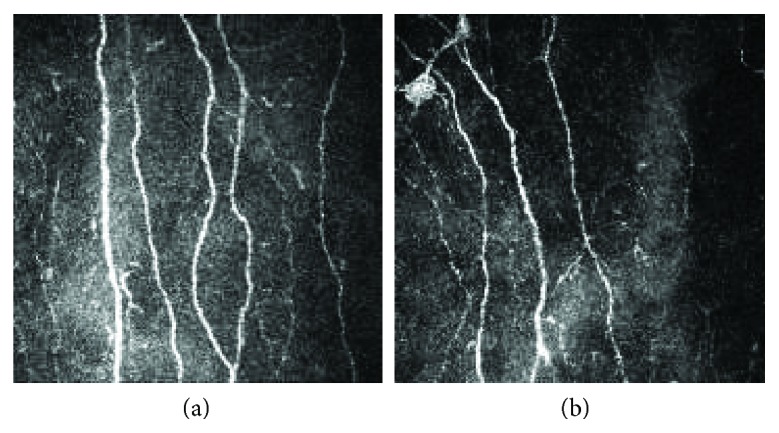
CCM images from a diabetic patient of group B at baseline (a) and follow-up (b).

Group B showed no significant change at one-year follow-up and remained with an average HbA1c ≥ 7.0% (from 8.55 ± 1.57% to 8.79 ± 1.05%, *P* = 0.527). In group B, metabolic indexes (weight, SBP, DBP, TC, TG, LDLC, and HDLC) were similarly unchanged. For DPN assessment, TCSS showed no significant change. SSNCV was significantly lower at one-year follow-up than baseline (47.93 ± 7.20 m/s to 44.67 ± 6.43 m/s, *P* = 0.024), while all other nerve conduction velocities remained unchanged. All three corneal nerve parameters measured (CNFD (17.19 ± 5.31 n/mm^2^ to 15.67 ± 4.16 n/mm^2^, *P* = 0.001, change of −2.92 ± 2.96), CNBD (19.33 ± 12.82 n/mm^2^ to 14.23 ± 6.56 n/mm^2^, *P* = 0.033, change of −6.82 ± 11.65), and CNFL (11.16 ± 2.57 mm/mm^2^ to 9.90 ± 1.75 mm/mm^2^, *P* = 0.011, change of −1.71 ± 2.37)) decreased at one-year follow-up in group B (Figures 1 and 3).

We have retrospect all the patient's outpatient records; patients in group A had 152 times of outpatient records for diabetes in the past year in all, while patients in group B had 78 times of outpatient records for diabetes in all. This suggested that patients in group A have higher compliance (Table 3).

### 3.3. Factors Affecting the Changes of Corneal Nerve Parameters

Multiple linear regression and correlative analysis showed that the changes of corneal nerve parameters (CNFD, CNBD, and CNFL) had not shown significant correlations with the duration of diabetes, changes of weight, blood pressure, blood lipids, and HbA1c (*P* > 0.05).

## 4. Discussion

Longitudinal data from the Rochester cohort support the contention that the duration and severity of exposure to hyperglycemia are related to the progression and hence severity of neuropathy [17]. Interventional studies have examined large-fiber neuropathy in the evaluation of DPN; however, interventions assessed in these studies have failed to demonstrate improvement of DPN. It is currently unclear whether glucose control can improve diabetic neuropathy and affect its natural history. Recently, there has been increasing interest in the assessment of small-fiber neuropathy both as a method of detection of early-stage DPN and as an indicator of potentially regenerable nerves. A lifestyle intervention study [18] in patients with impaired glucose tolerance showed improvements in weight, blood lipid levels, blood pressure, and blood glucose levels after one year of intervention but failed to show improvements in vibration sense and electrophysiology. In the aforementioned study, the quantitative sudomotor axon reflex test and intraepidermal nerve fiber density (IENFD) test, which detect small-fiber nerve function and structure, revealed improvements after one year. IENFD is the gold standard for the diagnosis of small-fiber neuropathy; however, it is difficult to clinically adopt nerve or skin biopsies due to their invasive nature. As a noninvasive diagnostic method for small-fiber neuropathy, CCM has multiple advantages, including high sensitivity [19, 20], good repeatability [21, 22], and easy operation.

Studies of DPN in people with type 2 diabetes often have confusing results [7, 23] because metabolic risk factors, such as glucose, lipids, blood pressure, and BMI, have been shown to be related to the development of DPN [5]. Therefore, we sought to utilize CCM in a noninterventional study to observe the influence of glucose control on the natural history of DPN. At one year follow-up, 50% of diabetic patients had achieved good glucose control with HbA1c < 7% (group A), with the remainder failing to meet this mark (group B). At follow-up, although there were positive trends towards the improvement in blood pressure and blood lipids, there were no significant changes observed in metabolic indexes other than HbA1c in all diabetic patients. In group A, the assessment of neuropathy showed significant improvements compared to baseline only in CNFD and CNFL; there were no significant changes observed in TCSS and lower extremity NCV. Assessment of neuropathy in group B showed a significant decrease in SSNCV. Corneal nerve parameters examined in this group including CNFD, CNBD, and CNFL also showed a decrease at one-year follow-up.

Diabetic neuropathy is a complication of diabetic microangiopathy that is specifically related to glucose control but also related to multiple metabolic factors especially in type 2 diabetes. We observed improvement of corneal nerve parameters but not TCSS or NCV in patients with type 2 diabetes with good glucose control, indicating that the corneal nerve, which is reflective of small-fiber neuropathy, can highly sensitively detect the improvement of neuropathy. In addition to glucose control, positive trends towards the improvement in blood pressure and blood lipids especially in group A also contribute to the improvement of corneal nerve morphology. In patients with type 1 diabetes who underwent combined pancreas and kidney transplantation, one year following transplantation, among all diagnostic parameters used to evaluate DPN, only CNFL, CNFD, and CNBD showed improvements [21]. In a study comparing continuous subcutaneous insulin infusion (CSII) and multiple injections of insulin in type 1 diabetes, although glycemic control was similar among the two treatment groups after 24 months, improvements were observed in CNFL, CNFD, and CNBD only in the CSII group [24].

We did not find any correlations between changes in corneal nerve parameters and the disease course of type 2 diabetes, weight, SBP, DBP, blood lipids, or HbA1c. In a previous 24-month observational study, the decrease in HbA1c value was significantly associated with an increase in CNFD [25].

Limitations of the current study include the small size of the study sample and the lack of randomization. Larger randomized studies with active intervention are required to confirm our findings. Another limitation is that HbA1c only reflects the glycemic status of 3 months; in this observational study, we could not get HbA1c values of every person every 3 months, so we do not know how long were the patients in good glycemic control before there were changes in the corneal nerve morphology. Nevertheless, the present data suggest that CCM may be a convenient, noninvasive technique to assess the progression of nerve damage and potentially to assess the effects of therapeutic intervention in future clinical trials of human diabetic neuropathy.

## 5. Conclusion

The results of this study suggest that morphological repair of corneal nerve fibers can be detected when glycemic control improves. In vivo CCM could be a sensitive method that can be applied in future longitudinal or interventional studies on diabetic neuropathy.

## Figures and Tables

**Table 1:** Clinical characteristics of control subjects and patients with diabetes at baseline and one-year follow-up.

Parameter	Baseline		One-year follow-up		*P* value		
	Control, Ia (*n* = 12)	Diabetes, IIa (*n* = 32)	Control, Ib (*n* = 12)	Diabetes, IIb (*n* = 32)	Ia versus IIa^∗^	Ib versus Ia^†^	IIb versus IIa^‡^
Age, y	54.4 ± 12.7	56.9 ± 14.7	55.3 ± 12.8	58.1 ± 14.6	0.488	≤0.001	≤0.001
Sex (M/F)	6/6	18/14			—	—	—
Duration of diabetes, y	—	11.2 ± 9.2	—	12.4 ± 9.0			≤0.001
Weight, kg	63.8 ± 7.4	71.0 ± 16.5	63.4 ± 8.1	70.1 ± 14.5	0.155	0.692	0.318
BMI, kg/m^2^	23.15 ± 2.40	25.80 ± 4.78	22.9 ± 2.20	25.50 ± 4.14	0.075	0.631	0.328
SBP, mmHg	122.3 ± 11.8	137.8 ± 16.3	122.8 ± 11.4	133.3 ± 14.2	0.005	0.564	0.175
DBP, mmHg	73.5 ± 5.6	82.6 ± 8.0	74.0 ± 5.5	81.3 ± 10.6	0.001	0.551	0.396
TC, mmol/L	4.88 ± 0.67	4.52 ± 1.07	4.71 ± 0.59	4.59 ± 1.14	0.407	0.296	0.902
TG, mmol/L	1.25 ± 0.56	1.94 ± 1.82	1.21 ± 0.51	1.78 ± 1.57	0.177	0.819	0.483
LDLC, mmol/L	2.78 ± 0.64	2.69 ± 0.72	2.67 ± 0.61	2.56 ± 0.83	0.884	0.457	0.326
HDLC, mmol/L	1.51 ± 0.28	1.22 ± 0.33	1.42 ± 0.28	1.30 ± 0.31	0.008	0.107	0.058
HbA1c, %	5.36 ± 0.12	8.22 ± 1.67	5.38 ± 0.11	7.58 ± 1.47	≤0.001	0.275	0.058
TCSS	0.6 ± 0.7	5.2 ± 4.5	0.7 ± 0.7	5.4 ± 4.8	0.001	0.674	0.338
PMNCV, m/s	50.20 ± 2.84	44.67 ± 4.07	49.34 ± 2.36	43.43 ± 3.80	≤0.001	0.201	0.496
TMNCV, m/s	50.56 ± 3.15	44.24 ± 4.60	50.03 ± 2.39	42.46 ± 4.76	≤0.001	0.341	0.439
SPSNCV, m/s	55.44 ± 3.99	48.71 ± 7.36	54.98 ± 3.23	46.76 ± 7.20	≤0.001	0.491	0.049
SSNCV, m/s	53.69 ± 3.05	48.34 ± 7.18	52.63 ± 2.00	44.92 ± 6.21	0.017	0.156	0.146
CNFD, n/mm^2^	29.31 ± 4.31	18.71 ± 4.73	28.29 ± 3.38	19.12 ± 5.99	≤0.001	0.093	0.643
CNBD, n/mm^2^	42.19 ± 13.91	21.80 ± 14.67	39.11 ± 18.11	20.78 ± 12.98	0.003	0.279	0.667
CNFL, mm/mm^2^	17.96 ± 2.40	11.81 ± 2.46	17.15 ± 2.44	11.63 ± 2.72	≤0.001	0.191	0.737

Results are expressed as mean ± SD or counts for categorical variables. ^∗^Independent-sample *t*-test. ^†^Paired-sample *t*-test. ^‡^*χ*^2^ test.

**Table 2:** Clinical characteristics of patients with type 2 diabetes with improved HbA1c at one-year follow-up (group A) and consistently poor glycemic control at one-year follow-up (group B).

Parameter	Baseline		One-year follow-up		*P* value		
	Group A0 (*n* = 16)	Group B0 (*n* = 16)	Group A1 (*n* = 16)	Group B1 (*n* = 16)	A0 versus B0^∗^	A1 versus A0^†^	B1 versus B0^‡^
Age, y	56.1 ± 17.5	59.4 ± 11.	57.3 ± 17.4	60.8 ± 11.1	0.521	≤0.001	≤0.001
Sex (M/F)	9/7	9/7			—	—	—
Duration of diabetes, y	11.3 ± 11.1	10.4 ± 7.5	12.6 ± 10.9	11.5 ± 7.3	0.780	≤0.001	≤0.001
Weight, kg	72.0 ± 19.0	69.8 ± 15.7	70.3 ± 16.2	68.9 ± 14.1	0.233	0.201	0.940
BMI, kg/m^2^	27.36 ± 5.49	24.12 ± 7.69	26.73 ± 4.50	24.10 ± 3.25	0.063	0.207	0.909
SBP, mmHg	135.6 ± 15.4	137.4 ± 17.2	134.9 ± 13.5	134.8 ± 15.4	0.307	0.867	0.059
DBP, mmHg	82.7 ± 9.3	82.5 ± 8.6	81.4 ± 11.3	82.2 ± 10.8	0.846	0.552	0.486
TC, mmol/L	4.46 ± 1.23	4.51 ± 1.07	4.44 ± 1.15	4.54 ± 1.11	0.896	0.954	0.208
TG, mmol/L	1.83 ± 1.93	1.92 ± 1.71	1.31 ± 1.16	1.74 ± 1.47	0.671	0.190	0.367
LDLC, mmol/L	2.55 ± 0.78	2.70 ± 0.73	2.45 ± 0.80	2.54 ± 0.80	0.424	0.739	0.677
HDLC, mmol/L	1.26 ± 0.34	1.21 ± 0.31	1.39 ± 0.33	1.29 ± 0.30	0.996	0.073	0.491
HbA1c, %	7.78 ± 1.62	8.55 ± 1.57	6.52 ± 0.59	8.79 ± 1.05	0.268	0.005	0.527
TCSS	4.4 ± 4.4	6.1 ± 4.5	4.5 ± 4.8	6.3 ± 4.7	0.293	0.544	0.468
PMNCV, m/s	44.47 ± 4.10	44.62 ± 4.15	44.46 ± 4.33	43.39 ± 3.84	0.895	0.984	0.124
TMNCV, m/s	44.15 ± 4.86	44.12 ± 4.35	43.56 ± 4.86	42.54 ± 4.66	0.793	0.269	0.951
SPSNCV, m/s	50.42 ± 6.81	48.66 ± 7.48	50.14 ± 7.19	46.64 ± 7.21	0.261	0.287	0.056
SSNCV, m/s	48.87 ± 7.89	47.93 ± 7.20	47.28 ± 6.05	44.67 ± 6.43	0.982	0.293	0.024
CNFD, n/mm^2^	18.55 ± 5.25	17.19 ± 5.31	21.78 ± 6.13	15.67 ± 4.16	0.070	0.005	0.001
CNBD, n/mm^2^	21.76 ± 16.10	19.33 ± 12.82	26.19 ± 13.87	14.23 ± 6.56	0.349	0.122	0.033
CNFL, mm/mm^2^	11.62 ± 2.89	11.16 ± 2.57	13.04 ± 2.44	9.90 ± 1.75	0.137	0.029	0.011

Results are expressed as mean ± SD or counts for categorical variables. ^∗^Independent-sample *t*-test. ^†^Paired-sample *t*-test. ^‡^*χ*^2^ test.

**Table 3:** Correlation coefficients between corneal nerve parameters and other indexes.

Correlation coefficients (*r*)	Change in CNFD	Change in CNBD	Change in CNFL
Age	0.085	−0.220	−0.056
Duration of diabetes	0.036	−0.047	0.206
Change of Weight	−0.203	−0.199	
Change of SBP	−0.006	−0.126	−0.006
Change of DBP	−0.241	0.121	0.064
Change of TC	−0.146	0.000	−0.061
Change of TG	−0.031	0.01	0.034
Change of LDLC	−0.138	−0.084	−0.119
Change of HDLC	−0.042	0.180	0.110
Change of HbA1c	−0.127	0.200	0.077

## Data Availability

All data generated or analyzed during this study are included in this article and available from the corresponding author upon request.
